# Visual Loss, Homonymous Hemianopia, and Unilateral Optic Neuropathy as the Presenting Symptoms of Vertebrobasilar Dolichoectasia

**DOI:** 10.1155/2013/562397

**Published:** 2013-04-24

**Authors:** Panteleimon Mortzos, Torben Lykke Sørensen

**Affiliations:** ^1^Department of Ophthalmology, Copenhagen University Hospital Roskilde, 4000 Roskilde, Denmark; ^2^Faculty of Health Sciences, University of Copenhagen, 2200 Copenhagen, Denmark

## Abstract

Vertebrobasilar dolichoectasia (VBD) is a relatively rare disorder for which unfortunately there is no treatment. Here we describe a case of simultaneous pre- and postchiasmal visual pathway pathology secondary to a space occupying VBD. In addition our patient demonstrates one of the very few cases of VBD compression of the retrochiasmal pathway with no other cranial nerve involvement.

## 1. Introduction

Dolichoectasia refers to a diffuse dilatation of an artery (differentiating it from aneurysms) as well as marked elongation and tortuosity of the vessel. Intracranially, the most commonly affected area is the vertebrobasilar segment with carotid and middle cerebral artery ectasia occurring less often. 

Although vertebrobasilar dolichoectasia (VBD) is frequently asymptomatic, presenting symptoms vary from being secondary to ischemia, compression, or rarely vascular rupture [1, 2].

VBD has been described to cause ophthalmological symptoms, by direct compression of cranial nerves such as the trigeminal and facial and less frequently the abducens and oculomotor nerves [3, 4]. In addition, rare cases of direct compression by a VBD of the optic tract or the chiasm causing visual loss have also been previously reported [5, 6]. 

Here, we report a patient with right homonymous hemianopia due to direct compression by the ectatic basilar artery on the left optic track and compressive left optic neuropathy by secondary displacement of the adjacent left internal carotid artery.

## 2. Case Presentation

A 71-year-old man presented with gradual, progressive, and bilateral visual loss and reading difficulties over the last 6 months. His medical history was unremarkable, and he had no other symptoms apart from failing vision. He had no previous ophthalmic history apart from moderate amblyopia on the right eye (RE). The best corrected visual acuities were 20/40 RE and 20/100 left eye (LE), respectively. Automated refraction revealed anisometropic myopia of −5,25 sph RE and −3,75 sph LE.

On Ishihara colour test the patient identified 15/15 plates on the RE and 13/15 plates on the LE. Pupils were equal and normally reactive to light and near stimuli. There was no sign of a relative afferent pupillary defect (RAPD). Ocular motility and remaining cranial nerve examination was normal. Optic disc evaluation showed normal discs, central cupping of 0.4, with myopic peripapillary chorioretinal atrophy and a slight tilt of the right disc. 

**Figure 1:**
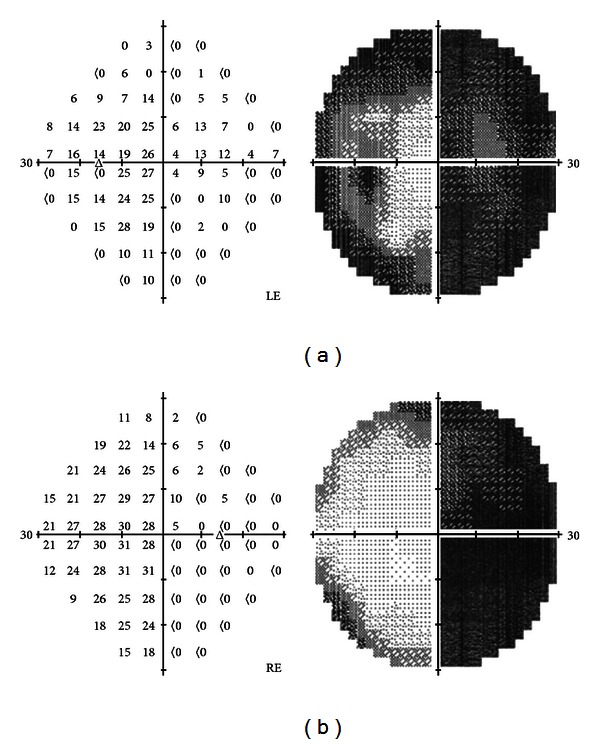
Visual field testing Humphrey 30-2. Left eye (LE) and right eye (RE), showing a right homonymous hemianopia with constriction of the remaining left hemifield on the LE.

Humphrey perimetry 30-2 revealed a dense right homonymous hemianopia with constriction of the remaining left hemifield in the LE (Figure 1).

**Figure 2:**
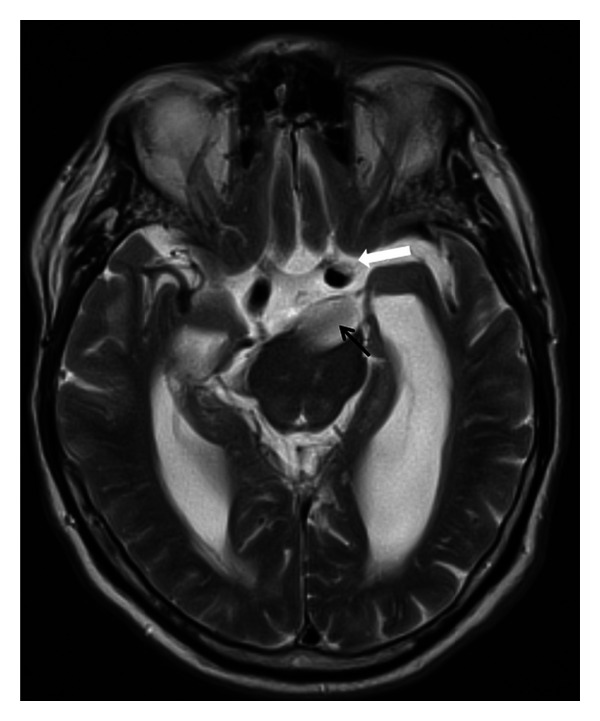
Magnetic resonance imaging (T2-weighted axial view). Ectatic basilar artery trunk compressing on the pons and the retrochiasmal pathway (black arrow depicting lumen of the basilar artery). Note the displaced internal carotid artery adjacent to the left optic nerve (white arrow).

**Figure 3:**
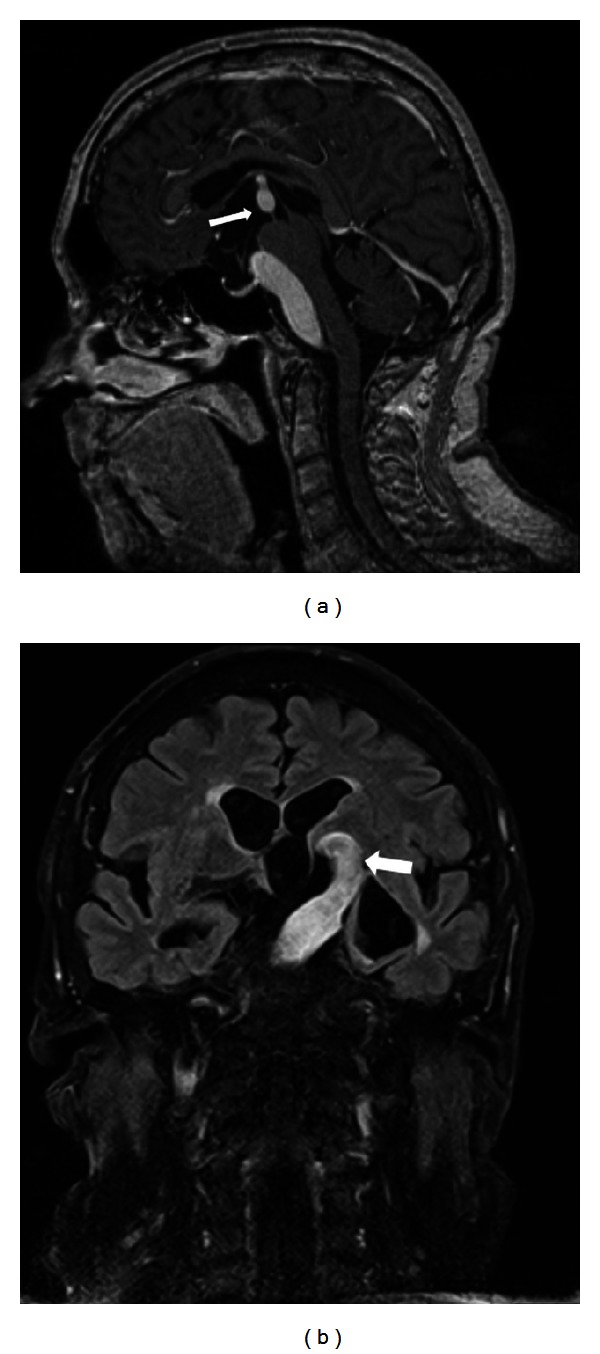
Magnetic resonance imaging (T1-weighted, contrast-enhanced). (a) Midline sagittal view (white arrow depicting dolichoectatic basilar artery elevating the floor of lateral ventricle). Note mass-effect on the brainstem. (b) Marked elevation of left optic tract (white arrow).

 Magnetic resonance imaging revealed a dilated right vertebral artery which continued as a severely ectatic (up to 1.5 cm) and elongated basilar artery with direct compression on the left optic tract. Those findings were diagnostic for VBD. There was mass-effect displacement of the left internal carotid artery which compressed the left optic nerve (Figures 2 and 3).

## 3. Discussion

To our knowledge, this is the first reported case of simultaneous pre- and postchiasmal visual pathway pathology secondary to a space occupying VBD. Our patient demonstrates one of the very few cases of VBD compression of the retrochiasmal pathway with no other cranial nerve involvement. In addition, due to the ectasias mass-effect, there is displacement of the left internal carotic artery, compressing the left optic nerve. We suggest that an expected RAPD of the left pupil is cancelled out by the simultaneously occurring RAPD of the right eye which is a consequence of the left optic tract lesion. 

VBD is a radiological diagnosis. The normal mean diameter of a basilar artery is 3.17 mm. Generally, ectasia is diagnosed if the diameter is over 4.5 mm [1, 7]. The severity of VBD depends on the calibre, the length, and tortuosity of the artery. The basilar artery and its bifurcation are located deep in the center of the cranial base with close proximity to central neurovascular structures. Posterior circulation ischemia, intracranial haemorrhage, and direct compression are the main risk factors to higher morbidity and mortality [7]. The most common long-term complications of dolichoectasia are ischemic stroke, brainstem compression, transient ischemic attack, hemorrhagic stroke, hydrocephalus, and subarachnoid hemorrhage [2]. The five-year mortality is approximately 30% [2]. 

Our patient remains free of any other neurological symptoms to this date. As surgical management is not recommended, his condition remains untreatable.
